# Lymph node identification in skin malignancy using indocyanine green transcutaneously study: Study protocol for a diagnostic accuracy study

**DOI:** 10.1097/MD.0000000000017839

**Published:** 2019-11-01

**Authors:** Ioana Lese, Jonathan Ian Leckenby, Adriano Taddeo, Mihai Constantinescu, Radu Olariu

**Affiliations:** a Department of Plastic and Hand Surgery, Inselspital; b Department for Biomedical Research, University of Bern, Bern, Switzerland; c Division of Plastic Surgery, Department of Surgery, University of Rochester Medical Center, Rochester, NY.

**Keywords:** ICG, malignant melanoma, Merkel cell carcinoma, NIRFI, sentinel lymph node

## Abstract

**Introduction:**

The incidence of malignant melanoma has been rising steadily over the past decades and Merkel cell carcinoma is a highly aggressive neuroendocrine skin tumor with a high mortality rate. Sentinel lymph node (SLN) biopsy is a recommended prognostic tool in primary cutaneous malignant melanomas of intermediate thickness and in all clinically node-negative Merkel cell carcinomas. The gold standard method for identification of SLNs is lymphoscintigraphy, which involves radioactive tracers. Indocyanine green-based near-infrared fluorescence imaging (NIRFI) has been also used for intraoperative SLN identification. We aim to evaluate the diagnostic sensitivity of the VisionSense VS3 NIRFI device for SNL identification. This device uses stereoscopic 3D high definition for both fluorescence and visible light imaging. Our hypothesis is that SLNs may be identified transcutaneously using fluorescent dye injections and NIRFI; therefore, obviating the need for lymphoscintigraphy in the future.

**Methods and analysis:**

lymph node identification in skin malignancy using indocyanine green transcutaneously is a prospective diagnostic sensitivity study conducted at the Department of Plastic and Hand Surgery at the University Hospital Berne, Inselspital, Switzerland. The study aims at recruiting 93 patients (start date September 2017) to compare the accuracy of VisionSense VS3 camera at identifying SLNs transcutaneously with the current gold standard, lymphoscintigraphy. Moreover, a secondary objective is to determine if anatomical location of the SLN and patient factors (eg, body mass index, age) have an impact on the ability of VisionSense to detect SLNs when compared with the same gold standard.

**Trial registration number:**

ClinicalTrials.gov NCT 03545334.

## 1 Introduction

Switzerland has the highest incidence of melanoma in Europe (19.2 per 100,000 per person-years).^[1]^ Worldwide, the incidence of malignant melanoma has been rising steadily over the past decades, according to the World Health Organization. Comparing cancer statistics from 2005 with data from 2015, the overall incidence of malignant melanoma increased by 56.2% during this period.^[2]^ Malignant melanoma is less common than other skin cancers, but at the same time has much higher mortality. The current treatment is dependent on the histological diagnosis following a biopsy and is determined primarily on tumor thickness (Breslow score), the rate the cancerous cells are dividing (mitotic rate), presence of ulceration, presence of regression, and age of patient. Initial treatment is through surgical excision with a safety margin of macroscopically normal skin surrounding the tumor. If the tumor thickness is more than 1 mm or more than 0.7 mm in association with high mitotic rate in younger patients, ulceration, regression or Clark level IV/V, the current cancer network guidelines suggest that the patient is offered a sentinel lymph node biopsy (SLNB) as this is most likely to be the first site any metastasis spreads to.^[3]^ Merkel cell carcinoma is a highly aggressive neuroendocrine skin tumor with a mortality of approximately 33% at 3 years.^[4]^ Because of the frequent lymphatic metastasis, a SLNB is highly recommended in all the patients to assess prognosis and guide treatment.^[5]^

SLNB is a staging tool for patients with clinically node-negative primary cutaneous malignancies and no evidence of distant metastasis. It is used to determine the histologic status of the nodes of the regional nodal basin(s) draining the primary site. If the sentinel lymph node (SLN) is negative, the rest of the nodes in the basin are presumed to be negative. The status of the SLN has been shown to be the strongest predictor of outcome in melanoma, in patients fulfilling the inclusion criteria mentioned above.^[6]^ SLNB detects Merkel cell carcinoma in one-third of patients whose tumors would have otherwise been clinically and radiologically under-staged and who may not have received treatment to the involved node bed. There is a significant benefit of adjuvant nodal therapy, but only when the SLNB is positive. Therefore, SLNB is important for both prognosis and treatment of Merkel cell carcinoma patients.^[7]^

The current gold standard technique to identify SLNs is to inject the radioisotope Technetium 99 m intra-dermally around the location of the primary tumor.^[8]^ The patient is then scanned while the radiotracer gives off energy in the form of gamma rays. The detection of these rays through a special camera gives then the location of the SLN at approximately 30 and 120 minutes. However, many surgeons favor the combination of radiocolloid with a blue dye to have 2 methods of identifying the SLN.^[9]^ However, there are concerns regarding the use of radioactive agents not only for the patients, but also for the medical staff.^[10]^

Indocyanine green (ICG) is a green dye reagent already used for hepatic function and cardiac output function.^[11]^ It can be used with near-infrared fluorescence imaging (NIRFI), having an absorption peak of 800 nm.^[12]^ The real-time fluorescence navigation with ICG for the identification of SLN in patients with malignant melanoma was first used in 2009.^[13]^ In the following years, this technique has been further researched and various publications are available on this subject.^[14,15]^ Even though other teams have tried to transcutaneously (“before skin incision”) identify SLNs with ICG and NIRFI, they concluded that ICG fluorescence technique is not reliable in patients with a high body mass index (BMI) or in patients with a primary tumor-draining in the axillary node field, and therefore a prior identification of the location of the SLN would be necessary with lymphoscintigraphy (LS). The SLN could; however, be easily identified “after skin incision” with a precision of 98.9%.^[16]^ Most papers cite a depth of 0.5 to 2 cm of the ICG that could be seen with the available NIRFI devices.^[17]^ Nevertheless, the current literature lacks clinical trials investigating the use of ICG and NIRFI in skin malignancies.

This study aims to evaluate a medical device that uses improved technology compared to previous studies: VisionSense VS3 (rumed GmbH, Lostorf, Switzerland). This device uses stereoscopic 3D high definition for both fluorescence and visible light imaging. Our hope is that by applying similar principles, SLNs may be identified transcutaneously using fluorescent dye injections and NIRFI; therefore, obviating the need for LS in the future.

### 2.1 Study objectives and diagnostic approaches

The primary objective of the lymph node identification in skin malignancy using indocyanine green transcutaneously (LIMIT) study is to investigate the diagnostic sensitivity of VisionSense VS3 NIRFI to transcutaneously identify SLNs in melanomas and Merkel cell carcinomas as effectively as LS, which is considered the current gold standard. The secondary objective is to determine if anatomical location of SLN and patient factors (eg, BMI, sex, age, etc) have an effect on the ability of VisionSense VS3 NIRFI to detect SLNs when compared with the same gold standard, LS.

### 2.2 Trial design and study setting

The LIMIT study is designed as a single-blinded, single-center, single-arm, not randomized diagnostic sensitivity study. The center in which the study is conducted is the Department of Plastic and Hand Surgery, University Hospital Berne, Inselspital (single-centre). The single-arm description refers to the fact that our study does not contain comparisons between different patients or interventions (no randomization). Since the surgeon will be blinded with regards to the number of lymph nodes (LNs) detected by the LS until after performing the procedure with ICG and NIRFI, the term “single-blinded” can be used to describe our study. The estimated study duration is 2 years and each patient will be assessed by both diagnostic approaches.

## 2 Methods and analysis

### 2.3 Guidelines and ethical conduct of study

The study will be carried out in accordance to the protocol and with principles enunciated in the current version of the Declaration of Helsinki, the guidelines of good clinical practice and the Swiss regulatory authorities’ requirements. We also designed our study in accordance with the “Standards for Reporting Diagnostic Accuracy Studies”.^[18]^

### 2.4 Patient population and eligibility criteria

Individuals fulfilling all of the following inclusion criteria are eligible for the study:

- Informed consent as documented by signature
- Patient must present with any of the following cancer types:
  ∘ Malignant melanoma with one of the following characteristics:
    (1) Breslow score ≥1 mm
    (2) Breslow score ≥0.7 mm associated with ulceration
    (3) Breslow score ≥0.7 mm associated with regression
    (4) Breslow score ≥0.7 mm associated with Clark Level IV/V
    (5) Breslow score ≥0.7 mm associated with mitotic rate ≥1/mm^2^ in young patients
  ∘ Merkel cell carcinoma

The presence of any one of the following exclusion criteria will lead to exclusion of the individual:

- Age <18 years
- Pregnancy and breastfeeding (pregnancy test to be performed for women of child-bearing potential, defined as women who are not surgically sterilized/hysterectomized, and/or who are postmenopausal for less than 12 months)
- Known allergy to ICG or iodine
- Previous chemotherapy, radiotherapy, or surgery to the LNs of interest
- Lack of capacity to provide informed consent
- Current enrolment in any other interventional study

### 2.5 Recruitment and consent

Patients who fulfill the inclusion criteria for the study will be sent an information packet detailing the background rationale of the study and the details of the surgical procedure. Before surgery a member of the study group will confirm the patient's willingness to participate in the study. If they agree, they will be formally consented regarding procedures, risks, and benefits of the study.

### 2.6 Criteria for withdrawal/discontinuation of participants

The participants are withdrawn from the study if they withdraw their consent, if the operation cannot take place anymore for medical reasons, if they are proven to have distant metastases following radiological staging or if there are no LNs on LS.

### 2.7 Outcome measures

Sensitivity on the number of LNs identified will be used as primary and secondary outcome measure.

The primary end-point is represented by the analysis of the case report forms (CRFs) collected after the surgeries: once the responsible surgeon has either identified or failed to identify a SLN transcutaneously, he/she is unblinded and the CRF is completed according to the comparisons made to the LS findings.

Secondary outcomes will be analyzed once the study period is completed. Data relating to the ability of SLNs to be successfully identified transcutaneously will be grouped anatomically or according to patient characteristics such as BMI, sex, and age and statistically analyzed to determine if certain anatomical regions or patient groups have different sensitivity rates.

Any patient who chooses to terminate the study early will receive the same postoperative (standard) care and follow-up as those included in the study.

### 2.8 Statistical analysis and sample size calculation

The statistical analysis will be performed by a statistician at the end of the study (ie, when all patients are included). Baseline characteristics will be reported as mean and standard deviation or median and interquartile range, and number and percentage of patients for continuous and categorical variables, respectively.

The sensitivity (ie, the proportion of NIRFI and LS positive LN among LS positive LN) will be reported with a 95% confidence interval (CI) and adjusted for multiple observations per patients using generalized estimating equations^[19]^ or the method by Rao and Scott.^[20]^ The number of LS-negative LNs that are NIRFI positive will be reported.

Data relating to the ability of SLNs to be successfully identified transcutaneously by NIRFI will be grouped anatomically and according to patients’ characteristics (eg, BMI, sex, and age) and afterward analyzed to determine if certain anatomical regions or patient groups have different sensitivity rates.

Only patients with positively identified LNs in LS will be included in the study and there will be no missing data from the LS. We do not expect any or only a small number of missing NIRFI data. However, patients with missing data in NIRFI will be excluded from the main analysis. For a sensitivity analysis, we will assume that all missing NIRFI results will be negative. The number of missing data will be reported together with the reasons behind their absence.

All of the patients will have at least 1 positive LN in LS, but there could also be cases with more than 1 LN per patient, increasing the precision of the sensitivity estimate. As it is not possible to make a reasonable assumption about the number of LNs per patient and the correlations of the measurements within a patient, we will recruit 93 patients. Sample size at the required absolute precision level for sensitivity was calculated with Buderer formula.^[19,20]^ We assumed a sensitivity of 60% (based on Namikawa et al – identification rate with ICG of 63.4% before skin incision^[16]^) and a maximum clinically acceptable width of the 95% CI of 10% (ie, 50%–70%), for a total of 93 LNs identified by LS.

### 2.9 Timeline

**Figure 1:**
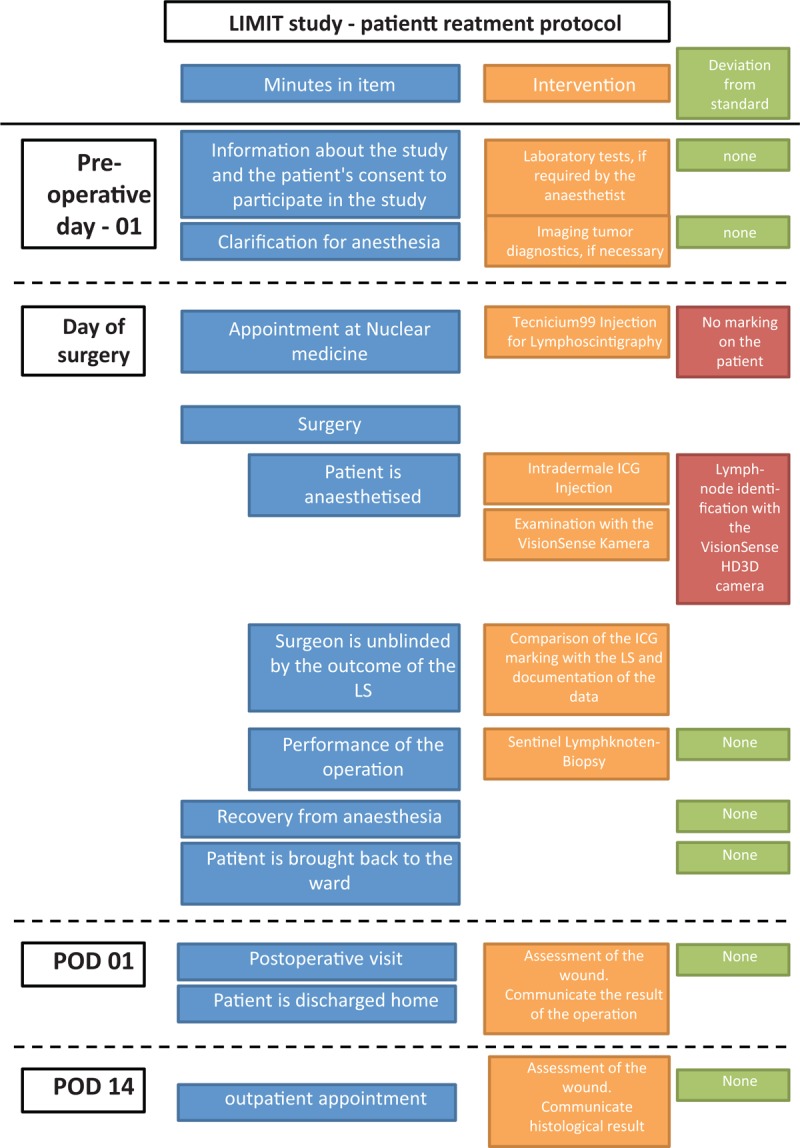
Timeline of a patient included in the study, including deviations from the standard.

At the beginning of the study, all involved personnel are introduced in their tasks. All the patients undergoing SLNB are checked to ensure they fulfill the inclusion/exclusion criteria of the trial. A patient information pack is sent to home address detailing the study and aims of the clinical trial. Figure 1 depicts the exact outline of a patient included in our study, including deviations from the standard. On admission, 1 day before the scheduled operation, a preoperative consultation explaining the surgical procedure including the risks and benefits of surgery takes place and the patient is consented for both the surgical procedure and for participation in the study. On the day of surgery, the patient attends a nuclear medicine appointment for the LS on the morning of the planned surgery. This will be performed in accordance with the standardized technique of injecting 4 small volumes (0.1–0.4 mL) of Technicium99 around the site of the primary tumor. The patient will then have scans taken at approximately 30 and 120 minutes following injection to locate the SLN(s), but he/she will not have the location of the SLN marked on his or her skin with permanent marker. The patient will be asked not to disclose the location of the detected SLN to the surgeon or any other member of the surgical team to ensure that the surgeon is blinded to the result and limit the degree of bias in the study.

After the patient is anaesthetized under full general anesthetic, the responsible surgeon will inject 0.2 to 0.4 mL of ICG intradermally around the site of the primary tumor. The surgeon will wait for a period of 5 to 15 minutes to allow for ICG to be absorbed and passed into the lymphatic system, and afterward, the VisionSense VS3 HD3D camera will be used to attempt to locate the SLN after confirming the camera is functioning correctly by commencing the scan at the site of ICG injection. The scan will proceed depending on the site of the primary tumor, with investigation of the presumed draining LNs. The identified LNs will be marked with a red marker and the locations documented in the CRF. Afterward, the nuclear medicine department will be telephoned and asked to upload the scans and radiology report of the LS onto the electronic system. The surgeon will visualize the location of the LS scan and locate them on the patient using a gamma probe using a blue permanent skin marker. The surgeon will ONLY perform the SLN biopsy on the LNs identified by LS. Any LNs identified by the VisionSense VS3 HD3D camera which do not correlate with the LS results will NOT be biopsied. The operation will be carried out in accordance with the standard operative procedure using a combined approach of gamma probe navigation and fluorescent imaging.

Once the patient has been re-admitted to the ward the responsible surgeon will make a routine postoperative visit of the patient and the surgical wounds will be inspected for any early operative complications. The patient will be informed of the surgical procedure and findings, including the results of the clinical trial. Any adverse events will be recorded in the CRF. A second postoperative consultation will take place on the day following surgery and the surgical wounds will be inspected again. Once the patient has fulfilled standard discharge criteria they will be prepared for discharge. A prescription for analgesia will be provided.

### 2.10 Data management and collection

The CRFs in this trial are implemented electronically using a dedicated electronic data capturing (EDC) system (REDCap). The EDC system is activated for the trial only after successfully passing a formal test procedure. All data entered in the CRFs are stored on a Linux server in a dedicated mySQL database.

Responsibility for hosting the EDC system and the database lies with the clinical trial unit Bern.

All data entered into the CRFs are transferred to the database using secure sockets layer encryption. Each data point has attributes attached to it identifying the user who entered it with the exact time and date. Retrospective alterations of data in the database are recorded in an audit table. Time, table, data field and altered value, and the person are recorded (audit trail).

A multi-level back-up system is implemented. Back-ups of the whole system including the database are run internally several times per day and on external tapes once a day. The back-up tapes are stored in a secure place in a different building.

### 2.11 Data monitoring and auditing

For quality control of the study conduct and data retrieval, the study site will be visited on-site by an appropriately trained and qualified monitor. Any findings and comments will be documented in site visit reports and communicated to the investigator and the sponsor as applicable. The investigator will support the monitor in his/her activities. Before the study start (first participant enrolled) a plan detailing all monitoring-related procedures will be developed.

All source data and relevant documents will be accessible to monitors and questions of monitors are answered during site visits.

Since this is a single-center investigator-initiated trial, the sponsor does not plan to perform any audits. If the competent authority (Swissmedic) or competent ethics committee performs an inspection on-site, staff will support the inspectors in their activities, study documentation, and the source data/source documents will be accessible to inspectors and study staff will answer questions from inspectors as needed. All involved parties must keep the participant data strictly confidential.

The number and type of adverse effects will be listed and reported. Also, any deviation will be communicated to the data monitoring committee and to the research team in a joint meeting.

An interim analysis will be performed after 60 examined patients to analyze how many LNs could be successfully identified transcutaneously by NIRFI until then and if there is a significant difference in the number of identified LNs with the standard methods.

### 2.12 Data protection: data access and confidentiality

Direct access to source documents will be permitted for purposes of monitoring and inspections. The investigator ensures anonymity of the patients; patients will not be identified by names in any documents leaving the study site. Signed informed consent forms and patient enrolment log will be kept strictly confidential to enable patient identification at the site.

Each LN is identified by a unique consecutive patient- LN identification number. A list with the identification numbers will be kept in the investigator site file at the study site, with access restricted to study personnel only.

### 2.13 Ethics and dissemination

The trial is registered in the ClinicalTrials.gov online trial database (NCT 03545334). The results of the trial will be published in subject matter related peer-reviewed scientific journals with a significant impact on the medical scientific community. The results of the trial will also be presented at the meetings of Swiss and international societies of Plastic Surgery and Dermatology. Since the use of the medical device is according to its intention there are no trade secret issues involved. Protocol modifications will require a formal amendment to the protocol which will be reported to the Competent Ethics Committee for approval.

### 2.14 Patient and public involvement

No patient involved.

## 3 Discussion

While the intraoperative detection rate of SLNs with ICG and NIRFI is 100%, the preoperative, transcutaneous identification rates have values of 23.8% in the axillary region and around 85% in the head and neck and groin area.^[16]^ Due to these low rates, the preoperative identification would still have to rely on the gold standard technique, the LS. However, the use of this technology is not without disadvantages: the use of radioactive agents is harmful not only for the patient, but also for the surgeon and staff.^[10]^

The medical device used in our study is the Visionsense VS3 – stereoscopic high definition visualization system. Its use is based on ICG fluorescence visualization transcutaneously with the device-specific infrared fluorescence visualization system. While other studies found the identification of SLNs “before skin incision” unreliable, we hope that the new technology with high definition infrared fluorescence scope and high definition 3D camera, as well as laser light source used to generate the fluorescence excitation illumination, will allow us to confirm our hypothesis and reduce/obviate the need for LS in the future in the future.

Because of the unpredictability of the number of patients, patient recruitment may be delayed and; therefore, the study period may not be adhered. Even though our sample size calculation might be underestimated, with some of the patients having more than 1 SLN, we believe that having more than 93 LNs will only increase our precision of the sensitivity estimate.

With our work, we hope to establish a new gold standard for the identification of SLN in cases of malignant melanoma and Merkel cell carcinoma. If our hypothesis proves true, we will be able to offer the patients a different technique for SLN identification, without the need for radioactive tracers; therefore, avoiding the risks associated with radiation.

### 3.1 Strengths and limitations of this study

This study aims at validating a new device for identification of SLNs to avoid in the future the use of radioactive tracers and their potential adverse effects for a common diagnostic procedure. The study will provide clear-cut data determining the safety and study design for randomized comparative studies between LS and NIRFI for SLN biopsies. A limitation of our study is the impossibility of determining specificity as the event of a false negative finding in LS is not possible

### 3.2 Trial status

The trial is currently actively recruiting patients. Completion of patient recruitment is expected for the beginning of 2020.

## Author contributions

**Adriano Taddeo**: design of the statistical analysis, the sample size calculation and the authoring of the corresponding sections

**Ioana Lese**: drafted the manuscript, primarily responsible for patient recruitment and monitoring the study at the ward, design of the statistical analysis, the sample size calculation and the authoring of the corresponding sections

**Jonathan Leckenby:** drafted the manuscript, authoring of multiple sections

**Mihai Constantinescu** (sponsor – mihai.constantinescu@insel.ch) provided clinical expertise for study design, revised the manuscript critically, and gave the final approval of the manuscript version to be published.

**Radu Olariu:** design and implementation of the study and the outline of the study protocol, conception of the general study approach but especially for definition of goals and outcome measures, timing and patient recruitment, revised the manuscript critically, and gave the final approval of the manuscript version to be published.

Ioana Lese orcid: 0000-0002-3007-097X.
